# Relationship Between Circulating Metabolic Hormones and Their Central Receptors During Ovariectomy-Induced Weight Gain in Rats

**DOI:** 10.3389/fphys.2021.800266

**Published:** 2022-01-05

**Authors:** Kaitlin E. Burch, Kelly McCracken, Daniel J. Buck, Randall L. Davis, Dusti K. Sloan, Kathleen S. Curtis

**Affiliations:** Department of Pharmacology and Physiology, Oklahoma State University Center for Health Sciences, Tulsa, OK, United States

**Keywords:** ghrelin, leptin, insulin, dorsal vagal complex, arcuate nucleus

## Abstract

Although increasing research focuses on the phenomenon of body weight gain in women after menopause, the complexity of body weight regulation and the array of models used to investigate it has proven to be challenging. Here, we used ovariectomized (OVX) rats, which rapidly gain weight, to determine if receptors for ghrelin, insulin, or leptin in the dorsal vagal complex (DVC), arcuate nucleus (ARC), or paraventricular nucleus (PVN) change during post-ovariectomy weight gain. Female Sprague-Dawley rats with *ad libitum* access to standard laboratory chow were bilaterally OVX or sham OVX. Subgroups were weighed and then terminated on day 5, 33, or 54 post-operatively; blood and brains were collected. ELISA kits were used to measure receptors for ghrelin, insulin, and leptin in the DVC, ARC, and PVN, as well as plasma ghrelin, insulin, and leptin. As expected, body weight increased rapidly after ovariectomy. However, ghrelin receptors did not change in any of the areas for either group, nor did circulating ghrelin. Thus, the receptor:hormone ratio indicated comparable ghrelin signaling in these CNS areas for both groups. Insulin receptors in the DVC and PVN decreased in the OVX group over time, increased in the PVN of the Sham group, and were unchanged in the ARC. These changes were accompanied by elevated circulating insulin in the OVX group. Thus, the receptor:hormone ratio indicated reduced insulin signaling in the DVC and PVN of OVX rats. Leptin receptors were unchanged in the DVC and ARC, but increased over time in the PVN of the Sham group. These changes were accompanied by elevated circulating leptin in both groups that was more pronounced in the OVX group. Thus, the receptor:hormone ratio indicated reduced leptin signaling in the DVC and PVN of both groups, but only in the OVX group for the ARC. Together, these data suggest that weight gain that occurs after removal of ovarian hormones by ovariectomy is associated with selective changes in metabolic hormone signaling in the CNS. While these changes may reflect behavioral or physiological alterations, it remains to be determined whether they cause post-ovariectomy weight gain or result from it.

## Introduction

As of 2019, 62% of women in the United States are considered to be either overweight or obese (Centers for Disease Control and Prevention [CDC], 2019), and weight gain is a particular problem among women of menopausal age (Ley et al., 1992; Ho et al., 2010; Coyoy et al., 2016). Clearly, overeating can contribute to weight gain, but regulation of feeding and body weight is a complex, integrated process that involves multiple hormones and neurotransmitters, and the involvement of numerous CNS pathways (Schwartz et al., 2000; Morton et al., 2006). Areas of particular interest within those pathways are the arcuate nucleus of the hypothalamus (ARC), which integrates adiposity signals. In turn, two distinct populations of cells within the ARC project to other areas of the hypothalamus, including the paraventricular nucleus (PVN). The transmission of these adiposity signals from the ARC to the PVN is thought to be responsible for decreased feeding. The PVN has reciprocal communication with the hindbrain dorsal vagal complex (DVC). The DVC, which includes the area postrema (AP), the nucleus of the solitary tract (NTS) and the dorsal motor nucleus of the vagus (DVX) sends and receives satiety signals to and from the gut. These central pathways, along with the associated hormones and neurotransmitters, are involved in the control of feeding and the regulation of body weight (Schwartz et al., 2000; Morton et al., 2006).

Notably, behavioral and physiological mechanisms that control feeding and body weight involve well-defined sex differences, as has been reported in studies of laboratory rodents (Wade and Gray, 1979; Asarian and Geary, 2013). Acyclic or ovariectomized (OVX) female rats eat more and gain more weight compared to intact (Tarttelin and Gorski, 1971; Blaustein and Wade, 1976; Geary and Asarian, 1999) rats, suggesting a role for the ovarian hormones, estrogen, and progesterone. Although some investigators have shown a role for progesterone in the control of body weight (Grueso et al., 2001; Stelmanska and Sucajtys-Szulc, 2014), most research has focused on estrogen. Indeed, the increased food intake and body weight gain in OVX rats is reversed by estrogen treatment (Tarttelin and Gorski, 1973; Blaustein and Wade, 1976; Geary and Asarian, 1999; Asarian and Geary, 2002), an effect thought to involve its modulation of the central actions of orexigenic hormones, like ghrelin, and anorexigenic hormones, like leptin and insulin (Brown and Clegg, 2010; Asarian and Geary, 2013). Ghrelin increases food intake (Kamegai et al., 2000; Tschop et al., 2000) and body weight (Tschop et al., 2000) by acting centrally (Nakazato et al., 2001; Brown and Clegg, 2010) at receptors including those localized to ARC, PVN, and DVC (Zigman et al., 2006). Circulating levels of insulin and leptin are proportional to the amount of body fat (Schwartz et al., 2000) and exert central actions to decrease food intake and body weight (Woods et al., 1979; Schwartz et al., 1992, 2000; Campfield et al., 1995; Morton et al., 2006) via receptors that are widespread throughout the CNS [insulin: (Zahniser et al., 1984; Werther et al., 1987], including ARC, PVN, and DVC [leptin: (Elmquist et al., 1998].

Increasingly, studies have focused on estrogen interactions with ghrelin, leptin, and insulin in food intake and body weight. Estrogen has been shown to decrease sensitivity to the orexigenic effects of ghrelin (Clegg et al., 2007; Butera et al., 2014). Furthermore, numerous studies have supported estrogen’s role in increasing sensitivity to produce an array of physiological and behavioral effects of both leptin (Clegg et al., 2006; Matyskova et al., 2010; Litwak et al., 2014) and insulin (Brussaard et al., 1997; Barros et al., 2006; Pratchayasakul et al., 2014; Yuan et al., 2015). These findings suggest that estrogen influences body weight by centrally-mediated responses to metabolic hormones. However, these effects require binding of metabolic hormones to their central receptors, and to date, the relationship between the reduction of ovarian hormones after ovariectomy, ovariectomy-induced weight gain, and expression of receptors for ghrelin, leptin, and insulin in the CNS remains incompletely understood. We hypothesized that ovariectomy changes central receptors for ghrelin, insulin, and leptin and, thereby, the dynamics between circulating levels of these hormones and their receptors. Accordingly, we assessed plasma levels of metabolic hormones and their associated central receptor levels during post-ovariectomy weight gain.

## Materials and Methods

### Animals

Female Sprague-Dawley rats (90 days of age, Charles River) were housed individually in plexiglass cages on a 12:12 dark:light cycle (lights on at 07:00) in a temperature controlled room (21–25°C) at Oklahoma State University-Center for Health Sciences and given *ad libitum* access to standard laboratory chow and water throughout the experiment. All procedures were approved by the Oklahoma State University—Center for Health Sciences Animal Care and Use Committee and were conducted in accordance with the National Institutes of Health Guide for the Care and Use of Laboratory Animals.

After acclimation to the colony room, rats were bilaterally OVX (*n* = 24) or sham OVX (*n* = 24). Ovariectomy was performed as previously described (Askew et al., 2015; Core and Curtis, 2017). Briefly, rats were orally administered 0.15 ml meloxicam (Meloxidyl, 1.5 mg/ml), then anesthetized with isoflurane (Covetrus, 3–5% induction; 1.5–3% maintenance), ovaries were removed, and the abdomen was closed. For the sham OVX group, the ovaries were located and isolated, then left intact, and the abdomen was closed. Rats were allowed to recover for 24 h, then body weight was obtained daily for the next 4 days.

One subgroup of rats (OVX: *n* = 8, sham: *n* = 8), was terminated on day 5 post-operatively. The remaining subgroups of rats were weighed weekly thereafter and terminated at day 33 (OVX: *n* = 8, sham: *n* = 8) or day 54 (OVX: *n* = 8, sham: *n* = 8) post-operatively. These time points were selected based on our previous publication (Curtis et al., 2018) to address any early post-surgical effects (5 days), and to correspond to the rapid weight gain after ovariectomy (33 days) and the established phase of weight gain after ovariectomy (54 days).

All rats were terminated between 10:00 and 12:00. Change in body weight was determined for each rat by subtracting body weight on the day of surgery from body weight on the day of termination.

### Tissue Sample Collection and Preparation

On the day of termination, rats were weighed, rendered unconscious with CO_2_ inhalation and then rapidly decapitated. Brains were collected on dry ice and stored at −80°C. Trunk blood and uteri were collected on ice. Blood was centrifuged at 1,400 × *g* for 15 min at 4°C, then plasma was collected and stored at −80°C. For each uterus, a 1 cm segment proximal to the uterine bifurcation was excised and weighed as an indication of the efficacy of ovariectomy (see Graves et al., 2011; Curtis, 2015).

For brain tissue preparation, ∼600–850 μm slices were taken from frozen brains at the levels of the DVC (^–^13.56 to ^–^14.40 from bregma), ARC (^–^2.64 to ^–^3.24 from bregma), and PVN (^–^1.32 to ^–^1.92 from bregma) (Paxinos and Watson, 2007). As described in our previous publication (Curtis et al., 2018), DVC, ARC, and PVN were identified via major landmarks (DVC—caudal inferior olivary nucleus, pyramidal tract, and central canal; ARC—caudal 3rd ventricle and median eminence, PVN—optic chiasm, anterior commissure, and rostral 3rd ventricle) as illustrated in Paxinos and Watson (2007).

Bilateral punches were taken from each area using a 1.0 mm Uni-Core punch (Ted Pella), collected into pre-weighed Eppendorf vials, and weighed to determine total tissue weight. Tissue was sonicated in 750 ml of cold PBS (3, 10 s pulses), and then centrifuged at 20,000 × *g* at 4°C for 20 min. Supernatants were then collected and stored at −80°C.

Total protein concentration of each supernatant was determined using a BCA protein assay as previously described (Curtis et al., 2018). Levels of leptin, ghrelin, and insulin receptors in brain regions were determined using commercially available ELISA kits (Wuhan Fine Biotech Co., Ltd., Rat Leptin Receptor ELISA Kit, Cat#ER1122; Cloud-Clone Corp., Rat Insulin Receptor ELISA Kit, Cat#SEA895Ra; MyBioSource, Rat Ghrelin Receptor Sandwich ELISA Kit, Cat#MBS9307176) according to manufacturers’ instructions. Plasma levels of leptin, ghrelin, and insulin were measured using commercially available ELISA kits (EMD Millipore Corp., Rat Leptin ELISA Kit 96-Well Plate, Cat#EZRL-83K; MyBioSource, Rat Ghrelin Sandwich ELISA Kit, Cat#MBS731169; EMD Millipore Corp., Rat/Mouse Insulin ELISA Kit 96-Well Plate, Cat#EZRMI-13K) according to manufacturers’ instructions.

To characterize the relationship between levels of receptors and circulating hormones, receptor: plasma hormone ratios were calculated for each rat by dividing the concentration of receptor for each hormone in each area of the brain by the plasma concentration of the corresponding hormone.

### Statistical Analysis

Data are presented as mean ± standard error of the mean (SEM). Data points that were more than 2 standard deviations from the mean were considered outliers and removed.

Data were analyzed by two-way ANOVA with treatment (OVX, Sham) and day (5, 33, 54) as factors. Pairwise comparisons of significant main effects or interactions were made using Fisher LSD tests. Significance was set at *p* < 0.05.

## Results

### Body Weight

Body weights of all groups over time are shown in the Supplementary Figure 1 to illustrate the pattern of weight gain (see also, Curtis et al., 2018). The OVX group clearly out-gains the Sham group, with weight on day 54 approximately 80 g greater in OVX than in Sham groups.

**FIGURE 1:**
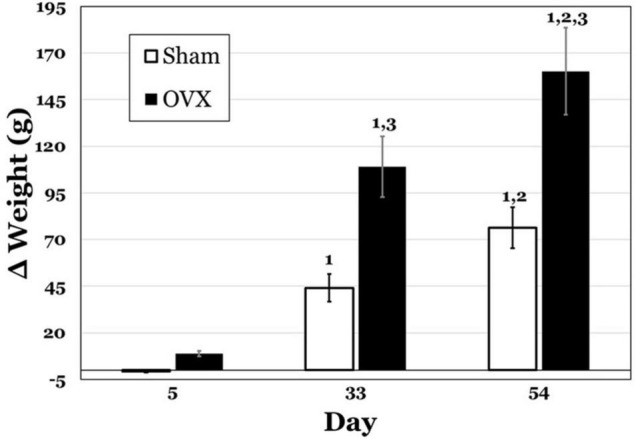
Change in body weight (g) in subgroups of ovariectomized (OVX; black bars) and Sham ovariectomized (Sham; white bars) rats that were terminated at day 5, 33, or 54 post-operatively. Data are presented as means ± SEM. 1 = significantly greater than day 5 for the same treatment group, 2 = significantly greater than day 33 for the same treatment group, 3 = significantly greater than Sham group at that specific day.

Figure 1 displays the change in body weights for both groups at 5, 33, and 54 days post-operatively. A two-way ANOVA revealed significant main effects of day [*F*(2,42) = 102.8, *p* < 0.0001] and treatment [*F*(1,42) = 64.36, *p* < 0.0001] and a significant interaction between day and treatment [*F*(2,42) = 11.51, *p* = 0.0001] for the change in body weights. Pairwise comparisons of the interaction indicated that weight gain in the OVX group was significantly greater on both day 33 and 54 than on day 5, as well as on day 54 than day 33 (all *p* values < 0.0001). While the Sham group also gained significantly more weight on day 33 and 54 than on day 5 (*p* = 0.0003, *p* < 0.0001, respectively), and significantly more weight on day 54 than on day 33 (*p* = 0.0071), weight gain in the OVX group was significantly greater than that in the Sham group on both day 33 and day 54 (both *p* values < 0.0001).

### Uterine Weight

**FIGURE 2:**
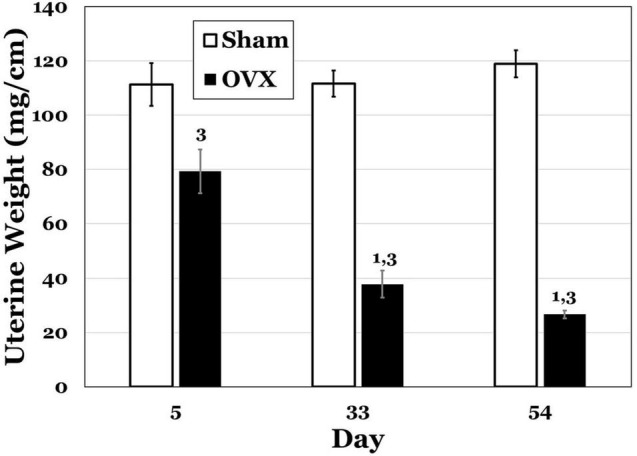
Uterine weights (mg/cm) at day 5, 33, or 54 post-operatively after ovariectomy (OVX: black bars) or sham ovariectomy (Sham: white bars). Data are presented as means ± SEM. 1 = s ignificantly less than day 5 for the same treatment group, 3 = significantly less than Sham group at that specific day.

In Figure 2, uterine weights for both groups are displayed, and the OVX groups exhibited overall lesser weights. A two-way ANOVA indicated significant main effects of day [*F*(2,42) = 8.230, *p* = 0.0010] and treatment [*F*(1,41) = 172.1, *p* < 0.0001] and a significant interaction between day and treatment [*F*(2,42) = 12.54, *p* < 0.0001] for uterine weights. Pairwise comparisons of the interaction showed significant differences for the OVX group. Specifically, day 5 was significantly greater than both day 33 and day 54 (both *p*s < 0.0001). There were also differences between the OVX and Sham groups at each day, with uterine weights in the OVX group significantly less at day 5 (*p* = 0.0007), day 33 (*p* < 0.0001) and day 54 (*p* < 0.0001). There were no differences in uterine weight over time for the Sham group.

### Ghrelin

#### Plasma Ghrelin

Levels of circulating ghrelin are shown in Table 1. A two-way ANOVA revealed no main effects of day or treatment and no interaction between day and treatment for plasma ghrelin levels.

**TABLE 1:** Circulating levels of Ghrelin, insulin, and leptin (pg/mg protein) at day 5, 33, or 54 post-operatively in ovariectomized (OVX) or sham ovariectomized (Sham) rats.

	Day	Sham	OVX
Ghrelin	5	3.591 ± 0.431	3.147 ± 0.470
	33	2.115 ± 0.630	4.261 ± 1.010
	54	4.036 ± 0.738	4.799 ± 0.958
Insulin	5	56.071 ± 3.381	89.063 ± 4.867^&^
	33	48.201 ± 5.290	76.255 ± 9.578^&^
	54	64.890 ± 5.472	70.978 ± 5.472
Leptin	5	79.566 ± 6.976	95.893 ± 12.690
	33	181.484 ± 36.616^@^	242.103 ± 32.880^@^
	54	176.612 ± 30.751^@^	374.072 ± 53.455^@#&^

*Data are presented as means ± SEM; separate two-way ANOVAs were performed for each hormone.*

^@^Significantly different than day 5 for the same group.

^#^Significantly different than day 33 for the same group.

^&^Significantly different than Sham group at that specific day.

#### Ghrelin Receptors, Ghrelin Receptor: Plasma Ghrelin Ratios (Figure 3)

*DVC*: A two-way ANOVA revealed no main effects of day or treatment and no interaction between day and treatment for ghrelin receptors in the DVC. Similarly, there were no differences in the ratio of ghrelin receptors to plasma ghrelin levels.

*ARC*: A two-way ANOVA revealed no main effects of day or treatment and no interaction between day and treatment for ghrelin receptors in the ARC, nor were there differences in the ratio of ghrelin receptors to plasma ghrelin levels.

*PVN*: A two-way ANOVA revealed no main effects of day or treatment and no interaction between day and treatment for ghrelin receptors in the PVN, or in the ratio of ghrelin receptors to plasma ghrelin levels.

**FIGURE 3:**
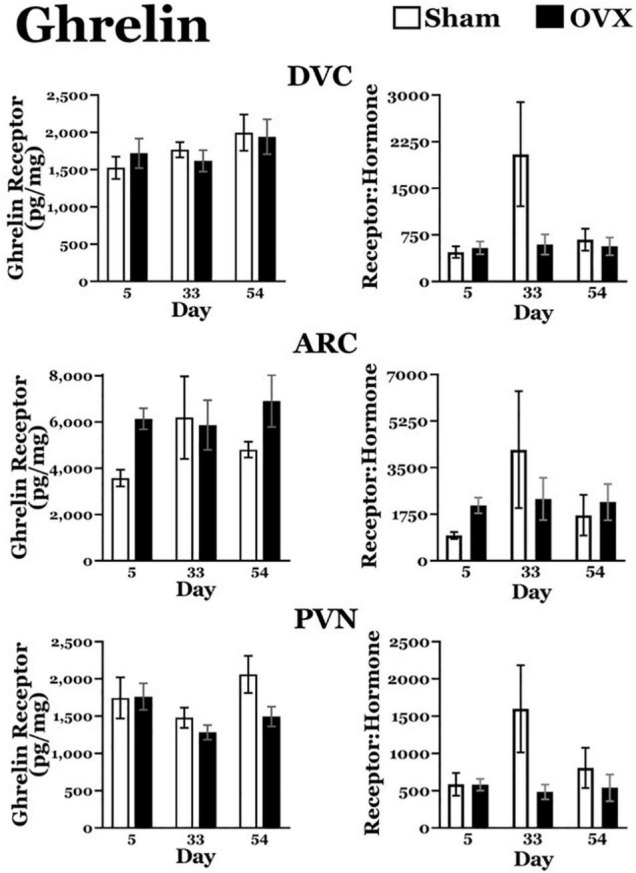
Ghrelin receptor levels (pg/mg protein) at day 5, 33, or 54 after ovariectomy (OVX: black bars) or sham ovariectomy (Sham: white bars). Top left: ghrelin receptor levels in the dorsal vagal complex (DVC). (Top right) Ratio of ghrelin receptor levels in the DVC to plasma ghrelin levels. (Middle left) Ghrelin receptor levels in the arcuate nucleus (ARC). (Middle right) ratio of ghrelin receptor levels in the ARC to plasma ghrelin levels. (Bottom left) Ghrelin receptor levels in the paraventricular nucleus (PVN). (Bottom right) Ratio of ghrelin receptor levels in the PVN to plasma ghrelin levels. Data are presented as means ± SEM; separate two-way ANOVAs were performed on each area. 3 = significantly different than Sham group at that specific day.

### Insulin

#### Plasma Insulin

Levels of circulating insulin are shown in Table 1. A two-way ANOVA revealed a significant main effect of treatment [*F*(1,40) = 16.86, *p* = 0.0002], with circulating insulin levels greater in the OVX group, overall. There was no main effect of day and no interaction between day and treatment for plasma insulin.

#### Insulin Receptors (Figure 4)

*DVC*: The OVX group displayed a downward trend in insulin receptor levels over time. A two-way ANOVA revealed no main effects of day or treatment for insulin receptors in the DVC, but a significant interaction between day and treatment [*F*(2,40) = 5.852, *p* = 0.0059]. Pairwise comparisons of the interaction showed that insulin receptors in the DVC of the OVX group were significantly greater on day 5 than on day 54 (*p* = 0.0027). Moreover, at day 54, insulin receptor levels in the OVX group were significantly less than those in the Sham group (*p* = 0.0059). There were no differences in insulin receptors in the DVC of the Sham group at any of the days examined.

*ARC*: A two-way ANOVA revealed no main effects of day or treatment and no interaction between day and treatment for insulin receptors in the ARC.

*PVN*: Similar to DVC insulin receptor levels, the OVX group exhibited a downward trend in insulin receptor levels in the PVN over day. A two-way ANOVA found no main effect of day, but a significant main effect of treatment [*F*(1,41) = 12.71, *p* = 0.009] for insulin receptors in the PVN. There also was a significant interaction between day and treatment [*F*(2,41) = 5.328, *p* = 0.0088] and pairwise comparisons of the interaction showed that insulin receptor levels in the OVX group receptor levels on day 5 were significantly greater than those on day 54 (*p* = 0.0096).

In addition, insulin receptor levels in the OVX group were significantly less than those in the Sham group on day 54 (*p* < 0.0001). At the same time, insulin receptors in the Sham group were significantly greater on day 54 than on day 33 (*p* = 0.005).

**FIGURE 4:**
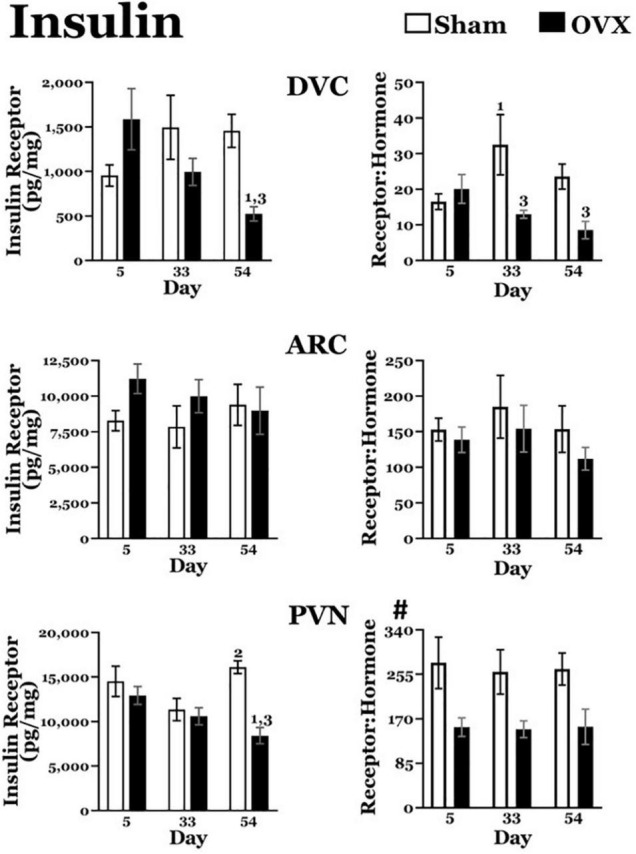
Insulin receptor levels (pg/mg protein) at day 5, 33, or 54 after ovariectomy (OVX: black bars) or sham ovariectomy (Sham: white bars). (Top left) Insulin receptor levels in the dorsal vagal complex (DVC). (Top right) Ratio of insulin receptor levels in the DVC to plasma insulin levels. (Middle left) Insulin receptor levels in the arcuate nucleus (ARC). (Middle right) Ratio of insulin receptor levels in the ARC to plasma insulin levels. (Bottom left) Insulin receptor levels in the paraventricular nucleus (PVN). (Bottom right) Ratio of insulin receptor levels in the PVN to plasma insulin levels. Data are presented as means ± SEM; separate two-way ANOVAs were performed on each area. 1 = significantly different than day 5 for the same treatment group, 2 = significantly different than day 33 for the same treatment group, 3 = significantly different than Sham group at that specific day. ^#^Significant main effect of treatment.

#### Insulin Receptor: Plasma Insulin Ratios (Figure 4)

*DVC*: The OVX group displayed a downward trend in the insulin receptor:plasma insulin over time. A two-way ANOVA revealed no main effect of day but a significant main effect of treatment [*F*(2,38) = 7.690, *p* = 0.0086] on the ratio of insulin receptors to insulin plasma levels in the DVC. There also was a significant interaction between day and treatment [*F*(2,38) = 3.431, *p* = 0.0427] and pairwise comparisons of the interaction showed that the ratio was significantly less in the OVX group than in the Sham group on both day 33 (*p* = 0.003) and day 54 (*p* = 0.0232).

In addition, the ratio was significantly greater on day 33 than on day 5 in the Sham group (*p* = 0.0163).

*ARC*: A two-way ANOVA revealed no main effects of day or treatment in the ratio of insulin receptors to plasma insulin levels in the ARC, nor an interaction between day and treatment.

*PVN*: Overall, the OVX group had a lower ratio of insulin receptors to plasma insulin levels in the PVN. A two-way ANOVA revealed a significant main effect of treatment [*F*(1,39) = 16.17, *p* = 0.0003) on the ratio of insulin receptors to plasma insulin levels in the PVN, but no effect of day, and no interaction between day and treatment.

### Leptin

#### Plasma Leptin

Levels of circulating leptin are shown in Table 1. A two-way ANOVA revealed significant main effects of day [*F*(2,41) = 18.34, *p* < 0.0001] and treatment [*F*(1,41) = 12.36, *p* = 0.0011] on plasma leptin levels, and a significant interaction between the two [*F*(2,41) = 4.480, *p* = 0.0174]. Pairwise comparisons of the interaction found an increase in circulating leptin levels over time in the OVX group, with levels on both day 33 and day 54 significantly greater than on day 5 (*p* = 0.0029, *p* < 0.0001, respectively), and levels on day 54 significantly greater than on day 33 (*p* = 0.0066). Leptin levels also increased over time in the Sham group, with levels on both day 33 and day 54 significantly greater than on day 5 (*p* = 0.0273, *p* = 0.0351, respectively). However, the increase in circulating leptin was more pronounced in the OVX group, with levels on day 54 significantly greater than those in the Sham group (*p* < 0.0001).

#### Leptin Receptors (Figure 5)

*DVC*: A two-way ANOVA revealed no main effects of day or treatment and no interaction between day and treatment for leptin receptors in the DVC.

*ARC*: A two-way ANOVA revealed no main effects of day or treatment and no interaction between day and treatment for leptin receptors in the ARC.

*PVN*: A two-way ANOVA revealed significant main effects of day [*F*(2,41) = 6.711, *p* = 0.0030] and treatment [*F*(1,41) = 10.01, *p* = 0.0029] on leptin receptors in the PVN, as well as a significant interaction between the two [*F*(2,41) = 3.753, *p* = 0.0319]. Pairwise comparisons of the interaction showed leptin receptor levels in the OVX group were significantly greater on day 33 than on day 5 (*p* = 0.0212). In the Sham group, leptin receptor were significantly greater on day 54 than those on day 5 (*p* = 0.0008) and day 33 (*p* = 0.0044). Finally, leptin receptors in the OVX group on day 54 were significantly less than those in the Sham group (*p* = 0.0005).

**FIGURE 5:**
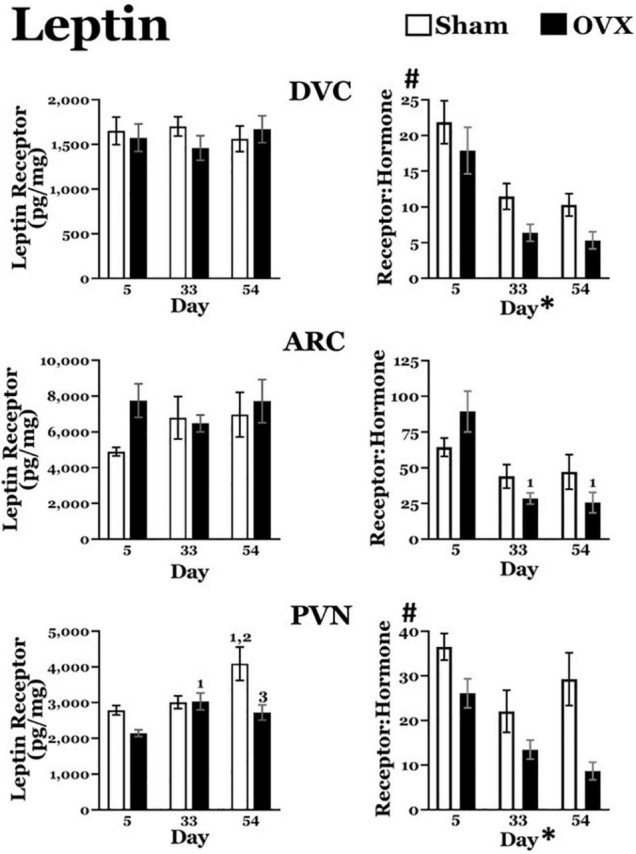
Leptin receptor levels (pg/mg protein) at day 5, 33, or 54 after ovariectomy (OVX: black bars) or sham ovariectomy (Sham: white bars). (Top left) Leptin receptor levels in the dorsal vagal complex (DVC). (Top right) Ratio of leptin receptor levels in the DVC to plasma leptin levels. (Middle left) Leptin receptor levels in the arcuate nucleus (ARC). (Middle right) Ratio of leptin receptor levels in the ARC to plasma leptin levels. (Bottom left) Leptin receptor levels in the paraventricular nucleus (PVN). (Bottom right) Ratio of leptin receptor levels in the PVN to plasma leptin levels. Data are presented as means ± SEM; separate two-way ANOVAs were performed on each area. 1 = significantly different than day 5 for the same treatment group, 2 = significantly different than day 33 for the same treatment group, 3 = significantly different than Sham group at that specific day. ^#^Significant main effect of treatment; *Significant main effect of day.

#### Leptin Receptor: Plasma Leptin Ratios (Figure 5)

*DVC*: Both sham and OVX groups displayed a decrease in the leptin receptor:plasma leptin ratio over time. A two-way ANOVA revealed significant main effects of both day [*F*(2,40) = 19.02, *p* < 0.0001] and treatment [*F*(1,40) = 7.060, *p* = 0.0113] on the ratio of leptin receptors to plasma leptin levels. A pairwise comparison of the main effect of day showed that, independent of treatment, the ratio was significantly greater on day 5 than on both day 33 and day 54 (both *p* values < 0.0001). There was no interaction between day and treatment.

*ARC*: The leptin receptor:plasma leptin ratio in the ARC also displayed a decrease over time in both groups. A two-way ANOVA revealed a significant main effect of day [*F*(2,39) = 11.13, *p* = 0.0002] on the leptin receptor:plasma leptin ratio, but no main effect of treatment. In addition, there was a significant interaction between day and treatment [*F*(2,39) = 3.255, *p* = 0.0488] and pairwise comparisons of the interaction showed that, in the OVX group, the ratio on day 5 was significantly greater than those on both day 33 (*p* = 0.0002) and day 54 (*p* < 0.0001). There were no differences in the ratio in the Sham group over time, nor did the two groups differ on any day.

*PVN*: The OVX group, but not the Sham group, displayed a downward trend in leptin receptor:plasma leptin ratio over time. A two-way ANOVA revealed significant main effects of both day [*F*(2,40) = 7.391, *p* = 0.0019] and treatment [*F*(1,40) = 17.49, *p* = 0.0002]. A pairwise comparison of the main effect of day showed that, independent of treatment, the ratio was significantly greater on day 5 than on day 33 (*p* = 0.0013) and on day 54 (*p* = 0.0027). There was no interaction between day and treatment.

## Discussion

It is well-known that ovarian hormones such as estrogen are involved in the control of food intake and body weight, and that this effect is, in part, attributable to the modification of central responses to metabolic hormones (Heine et al., 2000; Geary et al., 2001; Roesch, 2006; Santollo and Eckel, 2009; Thammacharoen et al., 2009; Rivera and Eckel, 2010; Santollo et al., 2010; Asarian and Geary, 2013; Sloan et al., 2018). It also is well-known that rats rapidly gain weight when ovarian hormones are eliminated by ovariectomy (Tarttelin and Gorski, 1971; Blaustein and Wade, 1976; McElroy and Wade, 1987; Geary and Asarian, 1999), though whether this procedure influences central receptors for metabolic hormones, or their relationships to circulating levels of those hormones has not been thoroughly investigated. In this study, we sought to determine whether the post-ovariectomy weight gain is associated with changes in the central receptors for ghrelin, insulin, and leptin, with changes in the circulating levels of these hormones, or with both.

Levels of insulin, ghrelin, and leptin in circulation depend upon various aspects of metabolic state and inform about fuel availability and/or stores, while serving as signals that affect body weight, feeding behaviors, and other metabolic processes (Schwartz et al., 2000; Butera et al., 2014). The specific effects produced by these hormones requires binding to receptors located in numerous areas, including central areas associated with the control of body weight, feeding, and metabolism such as DVC, ARC, and PVN (Schwartz et al., 2000). Thus, the impact of insulin, ghrelin, or leptin signaling depends not only on the amount of hormone present, but also on the number and location of receptors capable of binding the hormone. Accordingly, in addition to measuring insulin, leptin, and ghrelin receptors in DVC, ARC, and PVN, we also measured circulating levels of each hormone and then calculated the ratio between levels of receptors in each area and hormone in circulation. Clearly, other factors also are capable of influencing hormonal signaling. Nonetheless, our goal in calculating this ratio was to add another level to the characterization of hormonal signaling in these areas and any relationship to post-ovariectomy weight gain.

As expected, OVX rats gained considerable body weight (Tarttelin and Gorski, 1971, 1973; Blaustein and Wade, 1976; McElroy and Wade, 1987; Geary and Asarian, 1999; Asarian and Geary, 2002). In fact, both the amount of weight gained and the specific pattern of weight gain (substantial gain during the first 28–35 days after ovariectomy followed by a slower rate of gain during the next 20–25 days) were similar to that observed in our previous study (Curtis et al., 2018), and formed the basis for the time points examined in the present study. Importantly, despite the decreased rate of weight gain in OVX rats during the later phase of this study, differences in body weight between OVX and Sham groups persisted. It should be noted that we did not attempt to determine the stage of the estrus cycle in the Sham group at the time of testing. Although this may have contributed to some of the variability observed in measures of hormones and/or receptors, differences in uterine weight throughout the study suggests a sustained effect of ovarian hormones (Graves et al., 2011; Askew et al., 2015; Curtis, 2015; Core and Curtis, 2017) regardless of cycle stage. Moreover, we were able to detect differences in metabolic hormones and hormone receptors that depended on ovarian hormones—or on the weight gain associated with the absence of ovarian hormones. Some of these effects were specific to the area and/or the time frame examined.

### Ghrelin Signaling

Circulating levels of ghrelin did not change in either group, nor were ghrelin receptor levels altered in any of the areas examined. Thus, the ratio between ghrelin receptor to plasma ghrelin was unaffected in OVX rats. These findings are somewhat surprising, given the role of ghrelin to increase feeding and body weight (Kamegai et al., 2000, 2001; Tschop et al., 2000; Nakazato et al., 2001; Lv et al., 2018), and the finding that estrogen decreases the orexigenic effects of ghrelin (Clegg et al., 2007; Butera et al., 2014). We postulated that absent ovarian hormones, increased ghrelin signaling in ARC would stimulate greater food intake, while actions in the DVC and/or PVN would reduce metabolic rate due to their roles in autonomic function, and together these effects would contribute to post-ovariectomy body weight gain. This expectation was not met, suggesting that body weight gain in OVX rats does not involve central ghrelin signaling. However, it should be noted that all rats were terminated between 10:00 and 12:00, a time associated with low levels of activity and eating in these nocturnal animals. Circulating ghrelin levels typically peak shortly prior to eating (Sumithran et al., 2011), raising the possibility that, although basal levels of ghrelin measured in the quiet phase of the rats’ day:night cycle do not differ between the groups, pre-meal elevations in plasma ghrelin or in the central receptors in DVC, ARC, or PVN might be involved in the weight gain after ovariectomy. At the same time, we cannot rule out altered ghrelin signaling in other areas of the CNS involved in the control of feeding and/or body weight. Clearly, additional studies will be necessary to explore these findings further.

### Insulin Signaling

Circulating levels of insulin were greater in the OVX group throughout the study, which may reflect greater food intake and/or more frequent meals (Newsholme et al., 2014). Notwithstanding this difference, however, there were no differences in insulin receptors in the ARC, nor in the ratio between insulin receptor to circulating insulin in this area. In contrast, insulin receptor levels decreased in both the DVC and PVN in the OVX group by day 54, while insulin receptor levels in the PVN, but not the DVC, increased in the Sham group by day 54. As a result, the insulin receptor to plasma insulin ratios in both DVC and PVN were greater in the Sham group. The lack of effect of ovariectomy or body weight on insulin receptors (and on the ratio between insulin receptor to plasma insulin) in the ARC suggests that insulin signaling in this area does not play a role in the post-ovariectomy weight gain. On the other hand, for a given amount of circulating insulin, there were more receptors in both the DVC and the PVN in the Sham group. Thus, the lower insulin levels in the sham group may have nonetheless exerted effects that reduce feeding and enhance metabolism (Woods et al., 1979; Schwartz et al., 1992) due to increased receptors in the DVC and PVN, whereas the greater circulating insulin in OVX rats was less effective due to decreased receptors in both areas. One caveat to this interpretation, however, is that decreases in insulin receptors in OVX rats did not occur until day 54, after the initial phase of rapid weight gain in these rats. Thus, the differences in insulin signaling—at least in the DVC and PVN—may be associated with established obesity, rather than with the development of obesity.

### Leptin Signaling

Circulating levels of leptin increased in both groups, though this increase was more pronounced in OVX rats. Leptin levels increased in tandem with body weight (Considine et al., 1996) and were greater in the OVX group by day 54, the time point at which the body weight was greatest. Leptin receptor levels in the DVC did not change in either group but, owing to the less pronounced increase in circulating leptin in the Sham group, the leptin receptor to plasma leptin ratio was greater in that group, overall. Although leptin receptors in the ARC did not change in either group, the slight transient decrease in leptin receptors in the OVX group at day 33 in conjunction with the increasing levels of circulating leptin resulted in decreased leptin receptor to plasma leptin ratios at day 33 and thereafter. In the PVN, leptin receptor levels increased in Sham rats during the experiment. As a result, the leptin receptor to plasma leptin ratio in the PVN decreased over time in OVX rats, and was greater in the Sham group, overall. These findings suggest an overall influence of ovarian hormones on leptin signaling, raising the possibility that the combination of modest increases in circulating leptin levels and increased or sustained levels of leptin receptors in DVC, ARC, and PVN in the Sham group allowed leptin to continue to exert its effects on feeding, body weight, and metabolism (Friedman and Halaas, 1998). Interestingly, the decrease in leptin signaling in all three areas in OVX rats followed the general time course of the weight gain and elevated circulating leptin. Thus, early changes in leptin signaling in these areas in OVX rats may contribute to the weight gain and its metabolic consequences. While we focused on the direct effects of leptin in metabolism and weight gain in DVC, ARC, and PVN, it is important to note that leptin likely has actions in other central areas that may contribute to metabolism and/or weight gain. Moreover, leptin has been implicated in gastric emptying (Emond et al., 2001), which may indirectly affect feeding by altering signals of gastric distension. This effect likely occurs via receptors in the nucleus of the solitary tract (NTS), an integral part of the DVC. Moreover, the cardiovascular effects of leptin (Poetsch et al., 2020) also may involve receptors in the NTS. In either case, it remains to be determined whether these changes are secondary to the weight gain, rather than being causally related.

### Synthesis

**FIGURE 6:**
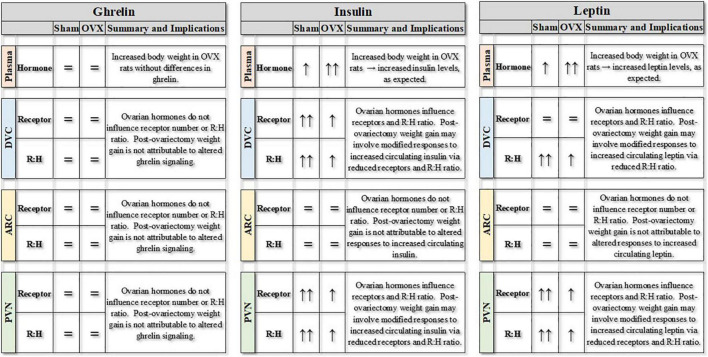
Summary of experimental findings for ovariectomized (OVX) or sham ovariectomized (Sham) rats. Plasma levels of ghrelin, insulin and leptin (pg/mg protein) are represented, along with receptors for those hormones (pg/mg protein) in three central nuclei involved in the control of feeding and body weight. Finally, the receptor:hormone ratios (R:H) for each hormone in each area also are represented. DVC, dorsal vagal complex; ARC, arcuate nucleus; PVN, paraventricular nucleus of the hypothalamus. = indicates no differences between groups or over time; ↑ indicates increase over time, with differences in the number of arrows indicating differences between groups.

In our previous study (Curtis et al., 2018), we reported that neuroimmune signaling in DVC, ARC, and PVN changed in site- and temporally-specific patterns during the weight gain after ovariectomy. Importantly, these effects occurred without concomitant changes in peripheral inflammatory factors or in free fatty acids. The present study expanded upon those findings to determine whether post-ovariectomy weight gain also may be associated with alterations in metabolic hormone signaling in these reciprocally-connected areas. As summarized in Figure 6, circulating levels of insulin and leptin, but not ghrelin, depended on both the absence of ovarian hormones and the development of the weight gain after ovariectomy. At the same time, there were inter-related changes in receptors for insulin, and leptin associated with post-ovariectomy weight gain in several interconnected brain areas involved in the control of feeding and body weight regulation. Additional studies may explore the relationship between hormonal and neuroimmune signaling in these areas, as well as the possibility of interactions between hormonal and neuroimmune signaling in other areas of the CNS that have been implicated in feeding and body weight regulation.

Arcuate nucleus, PVN, and DVC have been identified as areas in which receptors for estrogen have been localized. Thus, reduction of circulating estrogen levels after OVX may alter activity within these areas—and thereby affect feeding and body weight regulation. Interestingly, there are region specific differences in the subtypes. For example, the PVN contains primarily the ERβ subtype (Shughrue et al., 1997; Simonian and Herbison, 1997; Laflamme et al., 1998; Osterlund et al., 1998; Merchenthaler et al., 2004), while the ARC and the DVC contain primarily the ERα subtype (Shughrue et al., 1997; Laflamme et al., 1998; Merchenthaler et al., 2004; Vanderhorst et al., 2009; Kelly and Ronnekleiv, 2015). Indeed, estrogen effects on food intake and body weight are mediated by central estrogen receptors, with most evidence pointing to the ERα subtype (Geary et al., 2001; Roesch, 2006; Santollo and Eckel, 2009; Thammacharoen et al., 2009; Santollo et al., 2010). However, ERβ in the PVN also may play a role in the regulation of food intake and body weight via interactions with oxytocinergic neurons (Sloan et al., 2018).

Regardless of the subtype, however, the presence of estrogen receptors in these areas suggests a mechanism by which the presence or absence of estrogen may change the levels of receptors for ghrelin, insulin, or leptin. This effect may be direct, via the genomic effects of estrogen binding to its nuclear receptor in neurons that also contain receptors for ghrelin, insulin, or leptin. Alternatively, changes in metabolic hormone receptors in the ARC, PVN, or DVC may occur as the result of estrogen receptor-mediated actions at up-stream sites. An important caveat to these possibilities is that changes in the levels of receptors do not inform about receptor kinetics—and this difference may account for the discrepancy between the present observations of reduced insulin signaling in OVX rats and findings of reduced insulin sensitivity after estrogen treatment (Clegg et al., 2006). Finally, while estrogen has been shown to produce most effects on eating and body weight, we cannot rule out the possibility that progesterone or the absence thereof also plays a role (Grueso et al., 2001; Stelmanska and Sucajtys-Szulc, 2014 but see Asarian and Geary, 2006), perhaps by altering central receptors for insulin or leptin. Clearly, additional studies will be necessary to differentiate between these alternatives and to more conclusively determine the predominate receptor subtype in these effects.

An important goal in this study was to distinguish between changes in central receptors that occurred during the phase of rapid weight gain after ovariectomy from those that occurred in the face of established weight gain. In doing so, we selected specific time points after the surgeries and were able to detect differences in receptors for specific hormones as early as day 33 (i.e., during the rapid phase of post-ovariectomy weight gain) in specific areas. However, there was no temporal pattern of the observed changes that could provide information about the sequences in which those areas communicate. It is possible that a more exhaustive time course, in conjunction with evaluation of receptors for ghrelin, insulin, and leptin in other central areas (Zahniser et al., 1984; Werther et al., 1987; Elmquist et al., 1998; Zigman et al., 2006; see also Schwartz et al., 2000; Morton et al., 2006) would have provided the spatial and temporal resolution necessary to reveal more detailed information about the connectivity in these areas, but that was beyond the scope of the present study. In any case, it seems clear that most of the changes we observed were associated with increasing body weight. At present, whether the changes contribute to or result from increased body weight remain to be determined.

### Summary

The weight gain that occurs after ovariectomy was associated with region-specific differences in receptors for metabolic hormones and with changes in circulating levels of those hormones. Although our procedures did not reveal changes in ghrelin signaling, insulin signaling was decreased in the DVC and PVN of OVX rats and leptin signaling was decreased in ARC, PVN, and DVC, suggesting that reduced anorexigenic hormone responses contribute to the post-ovariectomy weight gain. Taken together, these findings further illustrate that the regulation of feeding and body weight involves a complex interplay of metabolic hormones and their receptors in interconnected CNS areas. In the absence of ovarian hormones, this multifaceted process may be disrupted at multiple levels—and with profound impact.

## Data Availability Statement

The raw data supporting the conclusions of this article will be made available by the authors, without undue reservation.

## Ethics Statement

The animal study was reviewed and approved by Oklahoma State University Center for Health Sciences Institutional Animal Care and Use Committee.

## Author Contributions

KB, KC, and RD conceived the study and designed the experiments. KB performed all the experiments with the assistance of KM, DB, RD, and KC. KB, KC, and DS analyzed the data with assistance from DB. KB, DS, and KC wrote the manuscript with input from DB, KM, and RD. All authors approved the submission of the final version.

## Conflict of Interest

The authors declare that the research was conducted in the absence of any commercial or financial relationships that could be construed as a potential conflict of interest.

## Publisher’s Note

All claims expressed in this article are solely those of the authors and do not necessarily represent those of their affiliated organizations, or those of the publisher, the editors and the reviewers. Any product that may be evaluated in this article, or claim that may be made by its manufacturer, is not guaranteed or endorsed by the publisher.

## Supplementary Material

Supplementary Figure 1 Time course of body weights in ovariectomized (OVX: black diamonds) or sham ovariectomized (Sham: white diamonds) rats. Both OVX and Sham groups were divided into subgroups that were terminated at day 5, 33, or 54 post-operatively. Data are shown for purposes of illustration (for comparison, see Curtis et al., 2018) and are presented as means ± SEM; some error bars are obscured by symbols.
