# Supplementary figures and images for: Relationship Between Circulating Metabolic Hormones and Their Central Receptors During Ovariectomy-Induced Weight Gain in Rats
Source: Front Physiol. 2022 Jan 5;12:800266. doi: 10.3389/fphys.2021.800266 (PMC8766843; doi:10.3389/fphys.2021.800266)

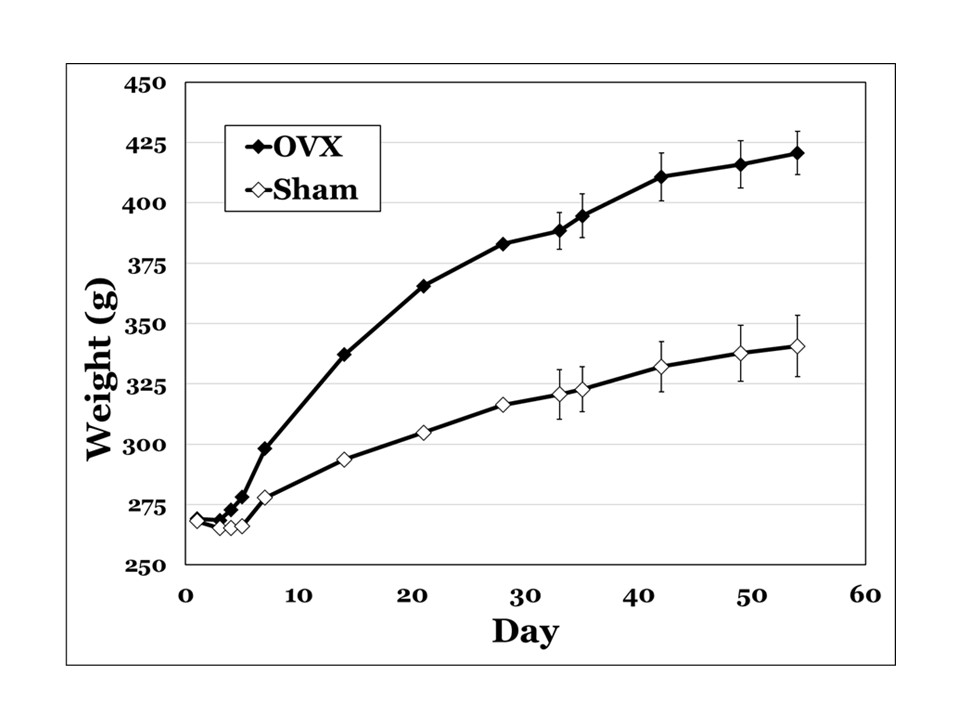

Supplement: Supplementary Figure 1 — Time course of body weights in ovariectomized (OVX: black diamonds) or sham ovariectomized (Sham: white diamonds) rats. Both OVX and Sham groups were divided into subgroups that were terminated at day 5, 33, or 54 post-operatively. Data are shown for purposes of illustration (for comparison, see Curtis et al., 2018) and are presented as means ± SEM; some error bars are obscured by symbols. [file Image_1.JPEG]
